# The Association of Toll-Like Receptor 4 Polymorphism with Hepatitis C Virus Infection in Saudi Arabian Patients

**DOI:** 10.1155/2014/357062

**Published:** 2014-08-10

**Authors:** Ahmed A. Al-Qahtani, Mashael R. Al-Anazi, Fahad Al-Zoghaibi, Ayman A. Abdo, Faisal M. Sanai, Mohammed Q. Khan, Ali Albenmousa, Hamad I. Al-Ashgar, Mohammed N. Al-Ahdal

**Affiliations:** ^1^Department of Infection and Immunity, Research Center, King Faisal Specialist Hospital & Research Center, Riyadh 11211, Saudi Arabia; ^2^Department of Microbiology and Immunology, Alfaisal University School of Medicine, Riyadh, Saudi Arabia; ^3^Liver Disease Research Center, King Saud University, Riyadh, Saudi Arabia; ^4^Molecular BioMedicine Program, Research Center, King Faisal Specialist Hospital & Research Center, Riyadh, Saudi Arabia; ^5^Department of Medicine, College of Medicine, King Saud University, Riyadh, Saudi Arabia; ^6^Division of Gastroenterology, Department of Medicine, King Abdulaziz Medical City, Jeddah, Saudi Arabia; ^7^Department of Medicine, King Faisal Specialist Hospital & Research Center, Riyadh, Saudi Arabia; ^8^Department of Gastroenterology & Hepatology, Prince Sultan Military Medical City, Riyadh, Saudi Arabia; ^9^Department of Pathology and Laboratory Medicine, King Faisal Specialist Hospital and Research Center, Riyadh, Saudi Arabia

## Abstract

Hepatitis C virus (HCV) is a single stranded RNA virus. It affects millions of people worldwide and is considered as a leading cause of liver diseases including cirrhosis and hepatocellular carcinoma. A recent study reported that TLR4 gene polymorphisms are good prognostic predictors and are associated with protection from liver fibrosis among Caucasians. This study aims to investigate the implication of genetic polymorphisms of TLR4 gene on the HCV infection in Saudi Arabian patients. Two SNPs in the TLR4 gene, rs4986790 (A/G) and rs4986791 (C/T), were genotyped in 450 HCV patients and 600 uninfected controls. The association analysis confirmed that both SNPs showed a significant difference in their distribution between HCV-infected patients and uninfected control subjects (*P* < 0.0001; OR = 0.404, 95% CI = 0.281–0.581) and (*P* < 0.0001; OR = 0.298, 95% CI = 0.201–0.443), respectively. More importantly, haplotype analysis revealed that four haplotypes, AC, GT, GC, and AT (rs4986790, rs4986791), were significantly associated with HCV infection when compared with control subjects. One haplotype AC was more prominently found when chronic HCV-infected patients were compared with cirrhosis/HCC patients (frequency = 94.7% and *P* = 0.04). Both TLR4 SNPs under investigation were found to be significantly implicated with HCV-infection among Saudi Arabian population.

## 1. Introduction

Hepatitis C virus (HCV) is a major global health problem, affecting more than 180 million people worldwide [1]. HCV genome is a single strand-RNA of a positive polarity and has a length of ~10 kb encoding a polyprotein of 3000 amino acids (aa) and flanked by 5′ and 3′ end untranslated region (UTR). Viral structural proteins (C, E1, and E2) are encoded by genes located at the N-terminal segment of the genome, while nonstructural (NS) proteins, encoded by genes located at the C-terminus of the open reading frame (ORF), include p7, NS2, NS3, NS4B, NS5A, and NS5B [2]. Based on phylogenetic studies, HCV was classified into six genotypes. However, it was reported that genotypes 1 and 4 are more resistant to the standard HCV treatment of pegylated interferon/ribavirin therapy [3].

Chronic HCV infection leads to variable degrees of hepatic inflammation with an increased risk of progression to cirrhosis and hepatocellular carcinoma (HCC) [4]. It is known that the host genetic background can influence the outcome of HCV infection. Several polymorphisms were found to be associated with HCV infection with many studies reporting the role of alleles, DQB1*0301 and DRB1*1101 in HCV clearance [5], while, other studies have found that variations in IL28B gene region to be significantly associated with spontaneous viral clearance, response to treatment with pegylated interferon (PEG-IFN)-based therapy [6, 7] and with the progression of the disease [8, 9]. HCV infection was implicated in a robust innate immune response including induction of interferon stimulated genes (ISGs) [10, 11]. The innate immune response is the first line of pathogenic defense which can be mediated by pathogen-derived signals such as lipopolysaccharide (LPS) from gram negative bacteria or viral DNA which are recognized by toll-like receptors (TLRs). TLRs belong to a highly conserved family of pattern recognition receptors that are capable of binding to pathogen associated molecular patterns (PAMPs), which upon activation lead to expression of inflammatory cytokines [12]. In a study conducted by Düesberg et al. [13], synthetic lipopeptide complexes of the HCV core protein were found to stimulate the innate immune response via TLR2 and TLR4. This was confirmed by other studies showing that HCV proteins, such as core and NS3, can activate human monocytes and macrophages via TLR2 [14, 15]. Other studies have also shown that HCV structural and nonstructural proteins interfere with the innate immunity signaling pathway through the interaction of ISGs [16–18].

TLR4, one of the most important and well-studied TLRs, is located on chromosome 9q32-33. TLR4 is known to recognize bacterial LPS but this receptor has also been found to recognize fusion protein from the respiratory syncytial virus (RSV) and the envelope protein of mouse mammary tumor virus (MMTV) [19, 20]. Reports have also shown that TLR4 can be stimulated by HCV nonstructural protein NS5A and thereby results in the secretion of IFNs and IL-6 from hepatocyte and B cells [21]. The activation of TLR2 and TLR4 signaling in hepatocyte leads to upregulation of proinflammatory cytokines and chemokines and recruitment of inflammatory cells to the liver [22].

Recently, TLR4 gene polymorphism Thr399Ile (rs4986791) was identified to be a good prognostic predictor for the development of cirrhosis in HCV infected patients [23, 24]. Also, it was shown that TLR4 SNPs, rs4986790 (D299G) and rs4986791 (T399I), are associated with protection from liver fibrosis, possibly through conformational changes of the protein, thereby affecting its interaction with other proteins [25].

In this study, we explored the association of the two cosegregating TLR4 single nucleotide polymorphisms (SNPs), rs4986790 and rs4986791, with HCV infection and the progression of the disease to liver cirrhosis and HCC in HCV infected Saudi patient.

## 2. Methods

### 2.1. Study Subjects

In this study, a total of 1050 subjects of Saudi origin, were recruited from three centers in Riyadh, Saudi Arabia, (King Faisal Specialist Hospital and Research Center, Riyadh Military Hospital and King Khalid University Hospital) between August 2007 and August 2010. These subjects include 450 HCV-infected subjects who were categorized as chronic HCV carriers (CHC), HCV-infected patients with liver cirrhosis (Cirr) who do not exhibit any clinical evidence of HCC, and patients who have progressed to HCC. Also, 600 randomly selected uninfected healthy controls that were serologically and PCR negative for HCV. The study protocol was approved by the institutional review boards of the three centers and conformed to the ethical guidelines of the 1975 Helsinki Declaration. Prior to their enrollment in this study, a written informed consent was obtained from all patients and their basic demographic data were recorded.

CHC subjects were diagnosed based on persistent detection of both HCV antibody and serum HCV RNA for at least six months with no signs of liver complications. Cirr was determined based on liver biopsy or diagnosed by the presence of at least two of the following: (a) radiological evidence (ultrasonography or computed tomography), platelet count < 90,000/L, or the presence of esophageal varices (demonstrated by endoscopy) and (b) at least two signs of liver dysfunction; Albumin level < 30 g/L, INR ≥ 1.5 or Bilirubin level > 35 *μ*mol/L. The diagnosis of HCC was made using noninvasive methods such as triphasic CT and/or MRI showing arterial enhancement of a liver lesion [26]. Patients diagnosed with other infections such as HBV or HIV were excluded from this study.

### 2.2. DNA Extraction and Genotyping of SNPs

Genomic DNA was extracted from peripheral blood mononuclear cells (PBMC) using Gentra Pure Gene kit (Qiagen, Hilden, Germany) according to the manufacturer's protocol. Samples from patients and control subjects were genotyped for the two polymorphic sites using TaqMan allelic discrimination assay with the 7900 HT Fast Real Time PCR System (Applied Biosystems, Foster City, CA, USA). All reagents required for the TaqMan assay including universal master mix, amplifying primers, and probes were purchased from Applied Biosystems (Foster City, CA, USA). The assay ID for rs4986790 is C_11722238_20 and for rs4986791 is C_11722237_20. One allelic probe was labeled with FAM dye and the other with the fluorescent VIC dye. PCR was run in the TaqMan universal master mix at a probe concentration of 20x in a 96-well format using 20 ng of genomic DNA in a total reaction volume of 25 *μ*L. The PCR reaction conditions were as follows: plates were heated to 50°C for 2 mins and then to 95°C for 10 mins; followed by 40 cycles of 95°C for 15 s and 60°C for 1.5 mins. The fluorescence intensity of each well in the TaqMan assay plate was read and the fluorescence data files from each plate were analyzed using automated allele-calling software (SDS 2.4) (Applied Biosystems, Foster City, CA, USA).

### 2.3. Statistical Analysis

Allelic and genotypic frequencies were estimated by direct counting and Pearson's chi-squared (*χ* ^2^) tests were utilized to determine the significance of the association between groups with a 2 × 2 contingency table. The results were expressed in terms of odds ratio (OR), 95% confidence intervals (95% CI) and *P* values. Odds ratios (OR) were calculated according to Woolf's method. When one of the critical entry was zero, Haldane's modification was applied to the Woolf equation using the formula: OR = [(*a* + 1/2) × (*d* + 1/2)]/[(*b* + 1/2) × (*c* + 1/2)], where *a*, *b*, *c*, and *d* represent affected individuals carrying the risk allele, nonaffected individuals carrying the risk allele, affected individuals carrying the nonrisk allele, and nonaffected individuals carrying the nonrisk allele, respectively. A *P* ≤ 0.05 was considered to be statistically significant. All statistical analyses were performed using SPSS software (version 17.0.0; SPSS, Chicago, IL, USA). The SNPs were tested for Hardy-Weinberg equilibrium (HWE) and a cut-off *P* value of 0.01 was set. Haplotype analysis and LD plots were generated using Haploview 4.2.

## 3. Results

All the samples from 450 HCV-infected patients and 600 control subjects were genotyped for the two SNPs in the TLR4 gene. Both SNPs, rs4986790 and rs4986791, were in Hardy-Weinberg equilibrium (HWE) within the study population and the minor allele frequency (MAF) was 0.08 for both SNPs (Table 1). The 450 patients included 354 chronic HCV carriers (CHC), 87 patients diagnosed with liver cirrhosis (Cirr), and 10 patients who have progressed to HCC. Of note, individuals carrying the minor genotypes, rs4986790-GG and rs4986791-TT, were found to be absent among the 450 HCV-infected patients recruited for the study.

A comparison between the HCV-infected patients with healthy controls revealed that the risk alleles rs4986791-T (OR = 0.298; 95% CI. 0.201–0.443, *χ* ^2^-value of 39.59 and *P* < 0.0001) and rs4986790-G (OR = 0.404; 95% CI. 0.281–0.581, *χ* ^2^-value of 25.25 and *P* < 0.0001) were found to be significantly distributed in HCV-infected patients (Table 2). The difference in the distribution of CC genotype of rs4986791 among patients (CC = 93%) and controls (CC = 78%) in comparison to the CT genotype (patients: 7%; controls: 22%), indicated that the individuals who carry the C allele recessively could be susceptible to HCV infection, as the T allele was found to have a significantly protective role in the dominant model with an OR of 0.274; 95% CI. 0.182–0.412 and a *P* value < 0.0001. A similar significant association was observed in the dominant model with an OR = 0.382; 95% CI. 0.262–0.557 and a *P* value < 0.0001 for rs4986790 with A as the dominant allele.

Since the development of HCC in HCV patients is as a result of cirrhosis of the liver, internal comparisons among the patient groups were done to determine whether the SNPs have any association with disease progression. However, these analyses revealed no statistical significance when CHC patients were compared to Cirr patients (Table 3) or Cirr/HCC patients (Table 4).

The two SNPs were found to be in a moderate linkage disequilibrium (LD) with *D*′ = 0.56,  *r* ^2^ = 0.307 and an LOD score of 50.47. When HCV patients were compared to healthy control group, four haplotypes were found to be significant - AC, GT, GC, and AT. Out of the four, rs4986790 (A)/rs4986791 (C) was found to be the most frequently occurring haplotype with a frequency of 0.888 and *P* < 0.0001 (Table 5). More importantly, the AC haplotype was found to be significant when CHC patients were compared with Cirr patients (frequency = 0.947; *P* = 0.031) (Table 6) or with Cirr/HCC patients (frequency 0.947 and *P* = 0.041) (Table 7).

## 4. Discussion

HCV causes significant health problems worldwide, as infection with the virus could lead to serious liver abnormalities including cirrhosis and HCC [4]. Variables such as old age, high ALT levels, high AFP levels, advanced fibrosis, virus genotype/subgenotype, and nonsustained virological response (non-SVR) are well-established risk factors for the severity of HCV infection, especially for the development of HCC [27–29]. However, host genetic factors have been shown to greatly influence the development of HCV-associated diseases [30]. Several studies have shown that variations in host genes could modulate HCV infection. For example, polymorphisms in IL28B gene were shown to influence the effectiveness of IFN-based therapy [31] and virus clearance [7]. Also, variations in other genes such as MICA [32], cytokines [33], chemokines [34], and DEPDC5 locus [35] were shown to be associated with disease progression in HCV-infected patients.

Ten TLRs were characterized in humans. TLR1, TLR2, and TLR6 were shown to interact with microbial lipoproteins, glycoproteins, and peptidoglycans. During the initial steps of an infection, these receptors participate in the recognition of such microbial antigens. Genomic DNA or RNA from several pathogens, including viruses, bacteria, and protozoa, is sensed and recognized by TLR9, which is also capable of recognizing modified forms of DNA such as unmethylated CpG islands. Single stranded RNA (ssRNA) and double stranded RNA (dsRNA) from viruses, such as West Nile virus (WNV), respiratory syncytial virus (RSV), HIV, and influenza virus, were found to be recognized by TLR3, TLR7, and TLR8. Although the exact function of TLR10 has not been fully elucidated, it is thought that it recognizes profilin-like molecules [36, 37]. TLR4 was the first TLR identified and it is a transmembrane receptor that has a critical role in innate immune system against infectious organisms [38]. TLR4 is expressed in several types of liver cells including hepatic stellate cell (HSC), hepatocytes, Kupffer cells, and biliary cells [21]. The mechanism of activation of TLR4 pathway is quite complex. It requires several auxiliary proteins (LBP and CD14) as well as a co-receptor called MD-2 [37]. MD-2 is a soluble protein with a large hydrophobic pocket and it was implicated in representing LPS to bind to TLR4 [39]. The MD-2 and TLR4 make a heterodimeric structure that interacts with LPS and results in the activation of the TLR4 signaling cascade [39, 40]. It was found that this heterodimer recruits two independent intracellular adaptor proteins (MyD88/TIRAP and TRIF/TRAM) which are involved in activation of two parallel signaling pathways that trigger the transcription of both proinflammatory cytokines and type I Interferon [41]. Of importance, TLR4 and MyD88-deficient mice developed significantly less liver tumors after feeding with the carcinogenic chemical, diethylnitrosamine. This observation suggests that TLR4-MyD88 signaling pathway plays a role in the development of liver tumor [42]. Although the main ligand for TLR4 is the bacteria endotoxin lipopolysaccharides (LPS) [12], it was also found that several HCV proteins, such as nonstructural protein 5A (NS5A), core, and NS3, stimulate TLR4 pathway [21].

Due to the importance of TLR4 in innate immunity, several studies have examined the role of TLR4 polymorphism in human diseases. Genetic variations in TLR4 gene have been linked to susceptibility to several infectious agents such as bacterial infection [43–45], viral infection [46, 47], malaria [48–50], hepatic fibrosis [25], and HCV-associated HCC [51]. Also, such variations have been reported to be associated with a wide variety of non-infectious diseases including diabetic neuropathy [52], atherosclerosis [53], rheumatoid arthritis [54], and premature birth [55]. TLR4 induction, signaling, and activation in numerous cancers such as breast cancer, colon cancer, ovarian, and prostate cancer were also identified [56]. Moreover, the induction of TLR4 was implicated as the molecular mechanism mediating liver damage and tumor formation in alcohol abused patients [57].

In this study, we examined the role of two nonsynonymous SNPs, rs4986790 (D299G) and rs4986791 (T399I), located at the third exon of the TLR4 gene on HCV infection. This was based on the observation that missense variations at these locations were implicated on the activity of TLR4 in inflammatory and fibrogenic signaling pathways [25]. Our findings showed that variations at these locations were found to be significantly associated with chronic HCV infection among Saudi nationals but not with HCV-induced liver abnormalities including cirrhosis or HCC. The minor allele carriers of both the SNPs (rs4986791-T and rs4986790-G) were comparatively lower in number among patients than in the control group, suggesting that variations at both SNPs could have a protective effect against HCV infection. Although, these results suggest that both genotypes could play a role in HCV infection, the clinical significance of this finding needs more investigation. Therefore, the probable protective effect of the two SNPs on HCV infection might be biologically possible from these facts but in order to confirm this, the present study must be replicated with more study subjects to rule out the possibility of population bias or the differences in ethnic background. Studies conducted in Spanish [51, 58] and Japanese [59] populations have reported no such significant associations, while in a study conducted among Caucasians, rs4986791-C was found to be associated with hepatic fibrosis progression, which was consistent with the present study [23]. In addition, the two SNPs were found to have a protective role among chronic hepatitis C infected Caucasians with liver fibrosis [24].

Interestingly, a study conducted by Tamura et al. [60] reported that HCV NS5A protein inhibited LPS-induced apoptosis of hepatocytes by downregulating the expression of TLR4 and thus might play a role in the pathogenesis of HCV infection [60]. In another study conducted by Ohto et al. [61], the two TLR4 SNPs were found to modulate the surface properties of TLR4 and hence might affect LPS binding to the TLR4-MD2 complex [61]. It has also been suggested that variation at these SNPs causes impairment of TLR4/MD-2 responses as a result of interfering with ligand-dependent dimerization [62]. The two SNPs have been found to be associated with hyporesponsiveness to LPS [63]. In a study conducted by Guo et al. [25], it was reported that the two TLR4 SNPs reduced the growth of murine hepatic stellate cells (mHSCs) and conferred an increased rate of spontaneous apoptosis in mHSCs [25]. On the contrary, it was also reported that the variation at amino acid 299 (D299G) causes inefficient recruitment of MyD88 and TRIF to TLR4, without disrupting the TLR4 expression, TLR4-MD2 interaction, or LPS binding and resulting in the impairment of TLR4 signaling pathway and thus playing a role in HCV pathogenesis [64].

According to NCBI HapMap database, the frequency of both SNPs investigated in this study has been documented in different ethnic populations. For rs4986790, the frequency was as follows: European (A = 0.967 and G = 0.033), Chinese Han (A = 1.000 and G = 0.000), Japanese (A = 1.000 and G = 0.000), Gujarati Indians in Houston (A = 0.902, G = 0.098), and African Yoruba (A = 0.967 and G = 0.033) populations. The allele frequencies observed among the Gujarati Indians were comparable to those observed among the Saudi Arabian population (A = 0.919 and G = 0.082). Similarly, the allele frequency for SNP rs4986791 is reported as follows: Europeans: (C = 0.955, T = 0.045), Chinese Han: (C = 0.988, T = 0.012), Japanese: (C = 1.000, T = 0.000), Gujarati Indians (C = 0.897, T = 0.103), and African Yoruba (C = 1.000, T = 0.000). The allele frequencies observed among the Saudi Arabian population (A = 0.922 and G = 0.078) was comparable to the Gujarati Indians and the European population.

Haplotype analysis revealed four significant haplotypes, AC, GT, GC, and AT between patient and control groups. However, the risk AC (rs4986790, rs4986791, resp.) haplotype was found to be more frequently occurring within the study population and comparatively more among the HCV-infected group than in controls, which accentuates the results of our study that the rs4986790-G and rs4986791-T alleles could have a protective role in HCV infection.

## 5. Conclusion

Both the TLR4 missense SNPs used in this study, rs4986790 and rs4986791 were found to be associated significantly with HCV-infected patients in the Saudi Arabian population. However, no significant association was found with advanced HCV-associated diseases such as cirrhosis and HCC.

## Figures and Tables

**Table 1:** Information of SNPs in TLR4 gene.

Name	Position	ObsHET	PredHET	HWpval	MAF	Alleles
rs4986790	120475302	0.156	0.147	0.0551	0.08	A : G
rs4986791	120475602	0.154	0.144	0.017	0.078	C : T

**Table 2:** Genotypic distribution of TLR4 SNPs between healthy control subjects and HCV-infected patients.

SNPs	Genotype/allele distribution	Healthy control subjects	HCV-infected patients	OR (95% CI.)	*χ* ^2^	*P* value
rs4986791	CC	469 (78.1%)	418 (93.0%)	1.0000 (Ref.)		
	CT	130 (21.7%)	32 (7.0%)	0.276 (0.184–0.416)	41.90	**<0.0001**
	TT	1 (0.2%)	0 (0%)	0.374 (0.000–10.803)	0.39	0.531
	C	1068 (89.0%)	868 (96.4%)	1.0000 (Ref.)		
	**T**	132 (11.0%)	32 (3.6%)	0.298 (0.201–0.443)	39.59	**<0.0001**
	TT + CT versus CC			0.274 (0.182–0.412)	42.50	**<0.0001**
	TT versus CT + CC			0.444 (0.000–12.805)	0.26	0.609

rs4986790	AA	475 (79.2%)	408 (90.9%)	1.0000 (Ref.)		
	AG	123 (20.5%)	41 (9.1%)	0.388 (0.266–0.566)	25.39	**<0.0001**
	GG	2 (0.3%)	0 (0%)	0.233 (0.000–4.472)	1.05	0.305
	A	1073 (89.4%)	857 (95.4%)	1.0000 (Ref.)		
	**G**	127 (10.6%)	41 (4.6%)	0.404 (0.281–0.581)	25.25	**<0.0001**
	GG versus AG + AA			0.266 (0.000–5.110)	0.84	0.359
	GG + AG versus AA			0.382 (0.262–0.557)	26.40	**<0.0001**

Risk allele marked in *bold* letters.

**Table 3:** Genotypic distribution of TLR4 SNPs between chronic HCV patients (CHC) and patients with liver cirrhosis (Cirr).

SNPs	Genotype/allele distribution	Chronic HCV patients	Patients with liver cirrhosis	OR (95% CI.)	*χ* ^2^	*P* value
rs4986791	CC	332 (94.0%)	77 (89.5%)	1.0000 (Ref.)		
	CT	22 (6.0%)	9 (10.5%)	1.764 (0.781–3.982)	1.91	0.167
	TT	0 (0%)	0 (0%)	nan		
	C	686 (96.9%)	163 (94.8%)	1.0000 (Ref.)		
	**T**	22 (3.1%)	9 (5.2%)	1.722 (0.778–3.809)	1.84	0.175
	TT + CT versus CC			1.764 (0.781–3.982)	1.91	0.167
	TT versus CT + CC			nan		

rs4986790	AA	323 (91.8%)	75 (86.2%)	1.0000 (Ref.)		
	AG	29 (8.2%)	12 (13.8%)	1.782 (0.869–3.654)	2.54	0.111
	GG	0 (0%)	0 (0%)	nan		
	A	675 (95.9%)	162 (93.1%)	1.0000 (Ref.)		
	**G**	29 (4.1%)	12 (6.9%)	1.724 (0.861–3.452)	2.42	0.120
	GG versus AG + AA			nan		
	GG + AG versus AA			1.782 (0.869–3.654)	2.54	0.110

Risk allele marked in *bold* letters.

**Table 4:** Genotypic distribution of TLR4 SNPs in chronic HCV carriers (CHC) compared with both patients with liver cirrhosis (Cirr) and patients with HCC combined.

SNPs	Genotype/allele distribution	Chronic HCV patients	Patients with liver cirrhosis + HCC	OR (95% CI.)	*χ* ^2^	*P* value
rs4986791	CC	332 (94.0%)	86 (89.6%)	1.0000 (Ref.)		
	CT	22 (6.0%)	10 (10.4%)	1.755 (0.801–3.844)	2.02	0.155
	TT	0 (0%)	0 (0%)	nan		
	C	686 (96.9%)	182 (94.8%)	1.0000 (Ref.)		
	**T**	22 (3.1%)	10 (5.2%)	1.713 (0.797–3.682)	1.94	0.163
	TT + CT versus CC			1.755 (0.801–3.844)	2.02	0.155
	TT versus CT + CC			nan		

rs4986790	AA	323 (91.8%)	85 (87.6%)	1.0000 (Ref.)		
	AG	29 (8.2%)	12 (12.4%)	1.572 (0.770–3.211)	1.565	0.211
	GG	0 (0%)	0 (0%)	nan		
	A	675 (95.9%)	182 (93.8%)	1.0000 (Ref.)		
	**G**	29 (4.1%)	12 (6.2%)	1.535 (0.724–3.203)	1.49	0.222
	GG versus AG + AA			nan		
	GG + AG versus AA			1.572 (0.770–3.211)	1.565	0.211

Risk allele marked in *bold* letters.

**Table 5:** Haplotype association analysis between healthy control subjects and HCV-infected patients.

Haplotype blocks		Freq.	HCV patients, healthy control ratio counts	HCV patients, healthy control frequencies	*χ* ^2^	*P* value
rs4986790	rs4986791					
A	C	0.888	855.1 : 48.9, 1014.2 : 185.8	0.946, 0.845	52.75	3.79 × 10^−13^
G	T	0.047	25.0 : 879.0, 73.2 : 1126.8	0.028, 0.061	12.915	**0.0003**
G	C	0.033	16.3 : 887.7, 53.8 : 1146.2	0.018, 0.045	11.557	**0.0007**
A	T	0.032	7.7 : 896.3, 58.8 : 1141.2	0.009, 0.049	27.57	1.52 × 10^−7^

**Table 6:** Haplotype association analysis between chronic HCV carriers (CHC) and patients with liver cirrhosis (Cirr).

Haplotype blocks		Freq.	Liver cirrhosis, chronic HCV ratio counts	Liver cirrhosis, chronic HCV frequencies	*χ* ^2^	*P* value
rs4986790	rs4986791					
A	C	0.947	159.0 : 15.0, 677.8 : 32.2	0.914, 0.955	4.652	**0.031**
G	T	0.029	6.6 : 167.4, 18.9 : 691.1	0.038, 0.027	0.632	0.426
G	C	0.018	5.4 : 168.6, 10.2 : 699.8	0.031, 0.014	2.251	0.133

**Table 7:** Haplotype association analysis between chronic HCV carriers (CHC) and both patients with liver cirrhosis (Cirr) and patients with HCC combined.

Haplotypes block		Freq.	Liver cirrhosis + HCC, chronic HCV ratio counts	Liver cirrhosis + HCC, chronic HCV frequencies	*χ* ^2^	*P* value
rs4986790	rs4986791					
A	C	0.947	178.0 : 16.0, 677.8 : 32.2	0.917, 0.955	4.193	**0.041**
G	T	0.028	6.6 : 187.4, 18.9 : 691.1	0.034, 0.027	0.3	0.584
G	C	0.017	5.4 : 188.6, 10.2 : 699.8	0.028, 0.014	1.64	0.200
